# Association between intraoperative dexmedetomidine and all-cause mortality and recurrence after laparoscopic resection of colorectal cancer: Follow-up analysis of a previous randomized controlled trial

**DOI:** 10.3389/fonc.2023.906514

**Published:** 2023-03-30

**Authors:** Jingping Hu, Chulian Gong, Xue Xiao, Liubing Chen, Yihan Zhang, Xiaoyue Li, Yanting Li, Xiangyang Zang, Pinjie Huang, Shaoli Zhou, Chaojin Chen

**Affiliations:** Department of Anesthesiology, The Third Affiliated Hospital of Sun Yat-sen University, Guangzhou, China

**Keywords:** colorectal resection, dexmedetomidine, recurrence, survival, colorectal cancer

## Abstract

**Background:**

Dexmedetomidine (DEX) has been widely applied in the anesthesia and sedation of patients with oncological diseases. However, the potential effect of DEX on tumor metastasis remains contradictory. This study follows up on patients who received intraoperative DEX during laparoscopic resection of colorectal cancer as part of a previous clinical trial, examining their outcomes 5 years later.

**Methods:**

Between June 2015 and December 2015, 60 patients undergoing laparoscopic colorectal resection were randomly assigned to the DEX and control groups. The DEX group received an initial loading dose of 1μ/kg before surgery, followed by a continuous infusion of 0.3μg/kg/h during the operation and the Control group received an equivalent volume of saline. A 5-year follow-up analysis was conducted to evaluate the overall survival, disease-free survival, and tumor recurrence.

**Results:**

The follow-up analysis included 55 of the 60 patients. The DEX group included 28 patients, while the control group included 27 patients. Baseline characteristics were comparable between the two groups, except for vascular and/or neural invasion of the tumor in the DEX group (9/28 vs. 0/27, *p* = 0.002). We did not observe a statistically significant benefit but rather a trend toward an increase in overall survival and disease-free survival in the DEX group, 1-year overall survival (96.4% vs. 88.9%, *p* = 0.282), 2-year overall survival (89.3% vs. 74.1%, *p* = 0.144), 3-year overall survival (89.3% vs. 70.4%, *p* = 0.08), and 5-year overall survival (78.6% vs. 59.3%, *p* = 0.121). The total rates of mortality and recurrence between the two groups were comparable (8/28 vs. 11/27, *p* = 0.343).

**Conclusion:**

Administration of DEX during laparoscopic resection of colorectal cancer had a nonsignificant trend toward improved overall survival and disease-free survival.

**Clinical Trial Registration:**

http://www.chictr.org.cn/, identifier ChiCTRIOR-15006518.

## Introduction

Surgical resections are the major treatment for most solid tumors and are associated with patients’ long-term functionality and quality of life. Perioperative treatment has shown great potential for influencing postoperative outcomes of cancer patients. For instance, intraoperative local anesthetic infusion would increase cancer-specific mortality in colon resections (1), and propofol-based total intravenous anesthesia was associated with better overall survival compared to volatile anesthesia in oncological patients (2). However, the effect of different anesthesia methods and anesthetics on the long-term prognosis of oncological patients remains controversial (3–5).

In recent years, dexmedetomidine (DEX), a highly selective alpha2 adrenoceptor agonist, has been widely applied in clinical anesthesia settings, including in oncological patients (6–8). However, whether DEX is reasonably used in tumor resections remains controversial. Some recent investigations suggested that DEX could promote tumor cell proliferation (9–11), metastasis, and migration *in vitro* (12, 13), and even decrease the overall postoperative survival in oncological patients who underwent lung resections (14), whereas others found that DEX would attenuate tumor cell metastasis and progression in the perioperative period (15–17). Regarding these controversial reports, there is still a notable lack of high-quality clinical studies to clarify the effects of DEX on the long-term prognosis of cancer patients.

In a previous study, we examined the immediate effects of administering DEX during elective laparoscopic resection of colorectal cancer. The findings indicated that DEX improved postoperative gastrointestinal motility function and resulted in more stable hemodynamics throughout the surgery (18). In the current study, we conducted a 5-year follow-up analysis of the same cohort to investigate the impact of intraoperative DEX on long-term survival and tumor recurrence following laparoscopic resection of colorectal cancer.

## Methods

The present study was carried out in accordance with the Declaration of Helsinki and was approved by the Institutional Review Board of the Third Affiliated Hospital of Sun Yat-Sen University (approval number: [2015]02-95-02). The study was registered on the Chinese Clinical Trial Registry (www.chictr.org) on June 7, 2015 (registration number: ChiCTRIOR-15006518). The trial protocol, design, and short-term outcomes of the randomized double-blind clinical trial have been reported previously (18).

A total of 60 patients undergoing elective laparoscopic colorectal resection at the institution (The Third Affiliated Hospital, Sun Yat-Sen University, China) between June 2015 and December 2015 were randomly assigned to the DEX group and the control group. All patients were operated on under the same general anesthesia protocol as described previously (18). All surgical procedures were performed by the same surgical group. In the DEX group, a loading dose of DEX (1 μg/kg) was given before induction for 10 min, followed by continuous intraoperative infusion (0.3 μg/kg/h). The patients in the control group were given the same volume of saline instead. Patients who met the following criteria were excluded in our previous research: gastrointestinal motility disorder; abdominal surgery history; bradyarrhythmia including sick sinus syndrome, sinus bradycardia or atrioventricular block; long-term administration of sedatives; psychiatric or neurologic comorbidity; hepatic or renal dysfunction; or distant metastasis.

A follow-up analysis of postoperative mortality and tumor recurrence was conducted in November 2021. Medical records were extracted from the hospital information system (HIS), and telephone follow-ups were utilized to access patient information. Patients who had benign lesions, non-malignant polyps, or Stage IV metastatic disease were not included in the follow-up analysis. Survival rate was calculated from the date of surgery until the date of death resulting from any cause. The duration of disease-free survival was measured from the date of surgery to the date of recurrence or death due to any cause. All-cause mortality was defined as death by any cause, while cancer-specific mortality was defined as death due to metastatic progression. The types of recurrence were classified as locoregional or distant. The duration between the date of surgery and the date of recurrence was defined as the time to recurrence. Patients with no evidence of recurrence at the time of death were censored on the date of patients’ death, while patients who remained alive at the time of analysis were censored at the end date of the follow-up period.

Baseline characteristics compared between the two groups included age, gender, body mass index (BMI), American Society of Anesthesiologists Physical Status Classification (ASA grade), type of operation, American Joint Committee on Cancer (AJCC) stage, tumor pathology, and adjuvant chemotherapy treatment. To ensure that recorded postoperative complications up to 30 days after surgery were comparable in both groups, specific complications were defined according to the criteria shown in **Table 1** (19). The Clavien-Dindo classification system (20) was used to grade postoperative complications. If a patient experienced multiple complications, the highest grade was considered for analysis.

**Table 1:** Definition of perioperative complications.

Complications	Criteria
Cardiorespiratory	New-onset ischemia determined by electrocardiograms and plasma cardiac markers or new-onset arrhythmia in need of intervention; documented respiratory complication requiring antibiotic treatment, whether it is a clinical diagnosis hinted at by pyrexia, hypoxia, and sputum with a positive bacteriologic culture or a radiologic diagnosis
Wound	Documented erythema, discharge requiring antibiotic treatment, or wound dehiscence requiring closure
Anastomotic leak	Clinical or radiologic diagnosis requiring intervention
Urinary tract infection	Symptomatic infection and positive microbiology requiring treatment
Ileus	No flatus, abdominal distension, nausea, or vomiting prevented oral intake or required therapeutic use of the nasogastric tube
Urinary retention	Failure to pass urine that requires insertion of a urinary catheter

### Statistical analysis

Statistical analysis was conducted using SPSS 19.0 software (SPSS Inc., Chicago, IL). One-sample Kolmogorov-Smirnov test was performed to assess the normality of the quantitative data. Mean ± standard deviation (SD) was used to describe quantitative variables that followed a normal distribution, and the T-test was utilized to compare the differences between groups. Categorical data or data without normal distribution were presented as median (interquartile range) or counts and compared by Fisher’s exact test for categorical variables or otherwise by Mann–Whitney *U* test. Survival differences between groups were assessed by Kaplan-Meier curves and analyzed using the Mantel-Cox test. Statistical significance was defined *a priori* as a *p*-value < 0.05.

## Results

Out of the 60 patients, 55 from the previous randomized clinical trial were included in the follow-up analysis. Five subjects were excluded from the analysis because of metastatic tumor at the time of operation. In total, 28 patients received intraoperative DEX, while 27 received the same dose of saline.

### Baseline characteristics of the study population

Baseline characteristics between the two groups are listed in **Table 2**. Demographic characteristics were comparable between the two groups in age, gender, height, weight, BMI, ASA grade, operation type, tumor stage, and adjuvant chemotherapy treatment. The majority tumor type was adenocarcinoma at stages II or III. All patients underwent R0 resection. Tumor differentiation between the two groups was comparable. However, there was a significant difference between the two groups in vascular and/or neural invasion of the tumor, with more patients in the DEX group having vascular and/or neural invasion of the tumor (9/28 vs. 0/27, *p* = 0.002). There were no significant differences in either the grade or type of postoperative complications observed between the groups (**Table 3**).

**Table 2:** Subject characteristics.

	Control group (*n* = 27)	DEX group (*n* = 28)	*p*-value
Age (year; median (range))	60 (54–67)	59.5 (53–65)	0.376
Sex			
Male	14	11	0.349
Female	13	17	
Height (median (range))	160 (155–165)	162 (155.25–170)	0.146
Weight (mean (SD))	58.9 (7.4)	61.9 (11.8)	0.259
BMI (kg/m^2^; median (range))	23.0 (21.0–24.7)	23.5 (21.0–25.4)	0.711
ASA grade			
I	1	3	0.495
II	20	21	
III	6	4	
Operation			
Right hemicolectomy	6	8	0.690
Left hemicolectomy	6	6	
High anterior resection	2	0	
Low anterior resection	13	14	
AJCC stage			
I	2	6	0.327
IIA	13	14	
IIIA	1	0	
IIIB	4	4	
IIIC	5	1	
IV	2	3	
Tumor stage			
T1	2	2	0.383
T2	1	5	
T3	3	4	
T4	21	17	
Nodal stage			
N0	15	20	0.530
N1	7	5	
N2	5	3	
M stage			
I	25	25	1.000
II	2	3	
Tumor type			
Adenocarcinoma	27	25	0.248
Mucinous adenocarcinoma	0	3	
Adjuvant chemotherapy			
Yes	16	20	0.403
No	11	8	
Vascular and/or neural invasion			
Yes	0	9	0.002
No	27	19	
Tumor differentiation			
Carcinoma *in situ*	2	1	0.747
Poor	2	4	
Moderately	23	23	
High	0	0	
Tumor resection			
R0 resection	27	28	–
R1 resection	0	0	
R2 resection	0	0	

ASA, American Society of Anesthesiologists; BMI, body mass index; AJCC, American Joint Committee on Cancer; SD, standard deviation.

**Table 3:** Postoperative complications.

	Control group (*n* = 27)	DEX group (*n* = 28)	*p*-value
Complications	2	2	1.000
Complication grade^a^			
I	0	2	0.333
II	1	0	
III	0	0	
IV	0	0	
V	1	0	
Complication type			
Cardiorespiratory	0	0	
Wound	1	1	
Anastomotic leak	0	0	
Urinary tract infection	0	0	
Ileus	1	0	
Urinary retention	0	1	
Other	0	0	

a Complication grade definitions (20): Grade I: any deviation from the normal postoperative course without the need for pharmacologic treatment or surgical, endoscopic, and radiological interventions. Allowed therapeutic regimens are as follows: drugs as anti-emetics, antipyretics, analgesics, diuretics and electrolytes, and physiotherapy. This grade also includes wound infections opened at the bedside. Grade II: requiring pharmacologic treatment with drugs other than those allowed for grade I complications. Blood transfusions and total parenteral nutrition are also included. Grade III: requiring surgical, endoscopic, or radiological intervention. Grade IV: life-threatening complication (including central nervous system complications) requiring intermediate care/intensive care unit management. Grade V: death of a patient. Fisher’s exact test is used unless otherwise stated.

### Primary and secondary outcomes

**Figure 1:**
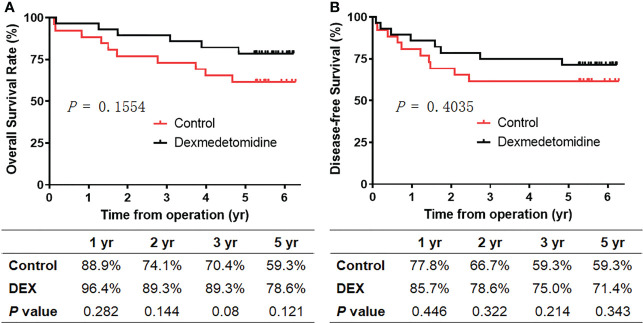
Survival of patients between the two groups. **(A)** Overall survival. **(B)** Disease-free survival. Kaplan–Meier curves showing overall survival and disease-free survival for patients receiving intraoperative dexmedetomidine (black line) or saline (red line).

By the time of analysis, the median duration of the follow-up was 5.3 years (1.72–5.58 years) in the control group and 5.47 years (5.24–6.03 years) in the DEX Group (**Table 4**). The primary outcome, the overall survival, is shown in **Figure 1**. The study did not demonstrate a statistically significant benefit for overall survival in 5 years, but rather a trend towards an increase in survival of the DEX group, which was demonstrated by relatively higher 1-year overall survival (96.4% vs. 88.9%, *p* = 0.282), 2-year overall survival (89.3% vs. 74.1%, *p* = 0.144), 3-year overall survival (89.3% vs. 70.4%, *p* = 0.08), and 5-year overall survival (78.6% vs. 59.3%, *p* = 0.121). Similarly, there was also a nonsignificant trend towards improved disease-free survival in DEX group in 1 (85.7% vs. 77.8%, *p* = 0.446), 2 (78.6% vs. 66.7%, *p* = 0.322), 3 (75.0% vs. 59.3%, *p* = 0.214), and 5 years (71.4% vs. 59.3%, *p* = 0.343).

**Table 4:** Mortality and cancer recurrence.

	Control group (*n* = 27)	DEX group (*n* = 28)	*p*-value
Follow-up time (year; median (range))	5.30 (1.72–5.58)	5.47 (5.24–6.03)	0.099
All-cause mortality	11	6	0.121
Cancer-specific mortality	10	5	0.110
Distant recurrence	8	4	0.205
Locoregional recurrence	2	3	1.000
Death or recurrence	11	8	0.343
Time from operation to recurrence (year; mean (SD))	1.08 (0.79)	1.11 (0.97)	0.950

Consistently, the all-cause mortality (6/28 vs. 11/27, *p* = 0.121) and cancer-specific mortality (5/28 vs. 10/27, *p* = 0.110) in the DEX group were relatively lower during the follow‐up period, though there were no significant differences (**Table 4**). Meanwhile, compared with the control group, there was a trend toward a lower rate of tumor distant recurrence in the DEX group (4/28 vs. 8/27, *p* = 0.205). The total rates of mortality and recurrence between the two groups were comparable (8/28 vs. 11/27, *p* = 0.343), as well as the rate of locoregional recurrence (3/28 vs. 2/27, *p* = 1.000). Moreover, there was no significant difference in the time from operation to recurrence between the two groups (1.08 (0.79) years vs.1.11 (0.97) years, *p* = 0.95).

## Discussion

This study tried to analyze the follow-up of the patients involved in a previously published randomized controlled trial who were operated on for colorectal cancer and who had DEX during the surgical procedure. We compared the long-term outcomes of patients who had DEX vs. those who had saline instead, after 5 years of follow-up. The results showed a nonsignificant trend toward improved overall survival and disease-free survival in the DEX group compared with the control group. The total rates of mortality and cancer recurrence between the two groups were comparable. However, the postoperative pathological results showed a significant difference in vascular and/or neural invasion of the tumor, there were more patients having vascular and/or neural invasion of the tumor in the DEX group. Patients receiving DEX had relatively lower all-cause mortality, cancer-specific mortality, and rate of distant recurrence, though not statistically different. However, the sample of the study was too small to get such results and conclusions. It would be more significant to wait and add more patients.

Being one of the most effective treatments for most solid tumors, surgical resection has been reported to potentially promote tumor metastases by different mechanisms, including the increased risks of micro-metastasis and the formation of new metastatic foci when shedding tumor lesions. Stress-related immunity suppression, the trauma-related release of growth factors to facilitate tumor cell proliferation, attenuated inhibition of angiogenesis after primary tumor removal, and the complex effect of anesthetics have also been reported to be involved (2, 21–24). The introduction of Enhanced Recovery After Surgery (ERAS) has prompted an increased focus among anesthesiologists on the impact of perioperative interventions on the long-term prognosis of cancer patients (25). There is growing evidence suggesting that perioperative care and different anesthetics can influence long-term oncological outcomes (26). For instance, it was suggested that patients who received propofol and sevoflurane in general anesthesia were associated with better overall survival than those who received desflurane alone (2). Although DEX has been shown to promote tumorigenesis in neurogliomas and lung carcinomas, breast cancer, and colon cancers (12, 27), others suggested that DEX could lower the tumor weight and tumor burden in xenograft mice with ovarian cancer (28), and repressed esophageal cancer cell proliferation *in vivo* (29). Despite the controversial *in vivo* results, the effect of DEX on long-term survival and tumor recurrence after laparoscopic resection of colorectal cancer has not been evaluated in the clinical setting.

Being a widely applied anesthesia adjuvant drug, administration of DEX has appeared to be associated with lower mortality in cardiac surgery and demonstrated a trend toward reduced cardiac complications in non-cardiac surgery (30–32). In a previous study conducted by our team, it was demonstrated that administering DEX during the intraoperative period improved the recovery of gastrointestinal motility function following laparoscopic resection of colorectal cancer (18). Vascular and neural infiltrations are known to be ominous prognostic factors in the tumor. The presence of vascular and/or neural invasion is associated with worse 5-year cancer-specific survival and worse 5-year overall survival in stages III and IV patients (33, 34). Although more patients in the DEX group had neurovascular invasion, there was no significant difference between the two groups in survival and mortality. Surprisingly, it presented a trend toward an increase in overall survival and disease-free survival in the DEX group. The study suggested that intraoperative administration of DEX may have potential benefits for the long-term prognosis of patients undergoing laparoscopic resection of colorectal cancer, which is consistent with the results of its recent application in uterine cancer surgery (35), but contradictory to what is biologically plausible based on some *in vivo* evidence (27, 36).

The contradictory findings could potentially be attributed to variations in the study subjects. It has been suggested that DEX may inhibit the hypothalamic-pituitary-adrenal (HPA) axis and reduce sympathetic activation (37). Surgical stress has been reported to activate the HPA axis and sympathoadrenal responses, which promote the expression of adrenoreceptors on T cells (38, 39), facilitate T cells to differentiate from Th1 into Th2 cells, thus altering the balance between the two subtypes, and result in inhibition of immune function (40, 41). Increasing evidence confirmed that administration with DEX was associated with improved postoperative immunosuppression, as reflected by the increased CD4^+^:CD8^+^ ratio and Th1:Th2 ratio (42, 43), and the results were also confirmed in the patients with colorectal cancer (38, 44).

Notably, we found the incidence of postoperative complications within 30 days after surgery in our study to be lower than in other reports (1, 26). We think this may be attributed to the superb technical skills of our gastrointestinal surgical team (45), who are devoted to applying total mesorectal excision with preservation of Denonvilliers’ fascia (iTME) in laparoscopic colorectal resection, which has shown to improve postoperative urogenital function (46).

This study has several limitations. Firstly, given that the initial randomized controlled trial was designed to detect postoperative intestinal function, The primary objective of the original study was not to assess long-term survival and cancer recurrence rates. Consequently, the sample size was limited, and the conclusions that can be drawn from this follow-up study are of restricted scope. As such, it should be noted that this study is exploratory in nature and serves to generate hypotheses for further investigation. A retrospective cohort study enrolling more patients who underwent laparoscopic resection of colorectal cancer could be conducted in the near future to confirm the current hypothesis. However, the inclusion and exclusion criteria, the dosage of dexmedetomidine, and the difference in surgical and anesthesia groups are all confounding factors that are difficult to control. Thus, it was difficult for us to expand the sample size for this study. A further multicenter randomized controlled study with a larger sample size would help to confirm the effects of dexmedetomidine on all-cause mortality and recurrence among patients who undergo laparoscopic resection for colorectal cancer. Secondly, we did not collect detailed information on the mediation and surgery history of the patients, and whether the patients in the control group also received DEX during the 5-year follow-up period was unclear; this might be another confounder. Despite its limitations, the initial randomized controlled trial design has enhanced the analysis in this study by ensuring subject randomization, which creates equivalent groups and minimizes the chance of significant confounding variables.

In summary, administration of DEX during laparoscopic resection of colorectal cancer had a nonsignificant trend towards improved overall survival and disease-free survival. The small sample size may limit statistically positive findings in the study. Studies with larger sample sizes should be developed to verify the results.

## Data availability statement

The original contributions presented in the study are included in the article/supplementary material. Further inquiries can be directed to the corresponding authors.

## Ethics statement

The studies involving human participants were reviewed and approved by Institutional Review Board of the Third Affiliated Hospital of Sun Yat-Sen University (approval number: [2015]02-95-02). The patients/participants provided their written informed consent to participate in this study.

## Author contributions

JH: data collection, data analysis, and write-up of the manuscript. CG and XX: data collection, analysis, and interpretation. LC: study design, data analysis, and interpretation. YZ, XL, YL and XZ: data collection and critical review of the manuscript. PH, SZ, and CC: study conception, study design, data analysis, and interpretation and critical review of the manuscript. All authors contributed to the article and approved the submitted version.
